# Promoter methylation status and expression of PPAR-γ gene are associated with prognosis of acute-on-chronic hepatitis B liver failure

**DOI:** 10.1186/s13148-015-0149-2

**Published:** 2015-10-28

**Authors:** Ze-Hua Zhao, Yu-Chen Fan, Qi Zhao, Cheng-Yun Dou, Xiang-Fen Ji, Jing Zhao, Shuai Gao, Xin-You Li, Kai Wang

**Affiliations:** Department of Hepatology, Qilu Hospital of Shandong University, Wenhuaxi Road 107#, Jinan, 250012 China; Institute of Hepatology, Shandong University, Wenhuaxi Road 107#, Jinan, 250012 China; Department of Gastroenterology, Provincial Hospital Affiliated to Shandong University, Jinan, 250012 China

**Keywords:** DNA methylation, Acute-on-chronic hepatitis B liver failure, Peroxisome proliferator-activated receptor gamma, Cytokines, Prognosis

## Abstract

**Background:**

Peroxisome proliferator-activated receptor gamma (PPAR-γ) has been demonstrated to be involved in anti-inflammatory reactions, but its role in acute-on-chronic hepatitis B liver failure (ACHBLF) is unclear. Therefore, DNA methylation patterns and expression level of PPAR-γ gene were detected in peripheral blood mononuclear cells (PBMCs) from 81 patients with ACHBLF, 50 patients with chronic hepatitis B (CHB), and 30 healthy controls, and the possible role of PPAR-γ in ACHBLF was analyzed.

**Results:**

We found that aberrant PPAR-γ promoter methylation was attenuated in ACHBLF patients compared with CHB patients and was responsible for the elevated PPAR-γ expression level, which was negatively correlated with total bilirubin and international normalized ratio. Plasma level of TNF-α and IL-6 in ACHBLF patients were higher than CHB patients and healthy controls and significantly reduced in unmethylated group. ACHBLF patients with PPAR-γ promoter methylation had poorer outcomes than those without. Correspondingly, PPAR-γ messenger RNA (mRNA) level was higher in survivors than non-survivors and gradually increased in survivors with time, while remained low level in non-survivors.

**Conclusions:**

Aberrant promoter methylation decline and PPAR-γ expression rebound occurred in ACHBLF compared with CHB and could improve prognosis of ACHBLF by negatively regulating cytokines.

**Electronic supplementary material:**

The online version of this article (doi:10.1186/s13148-015-0149-2) contains supplementary material, which is available to authorized users.

## Background

Hepatitis B virus (HBV) infection is one of the most serious public health problems, affecting more than two billion people worldwide. There are more than 350 million chronic carriers, 75 % of whom reside in the Asia Pacific region [1]. Reactivation of HBV infection is a major cause for acute deterioration of liver function manifesting as jaundice and coagulopathy, which is a severe life-threatening condition termed acute-on-chronic hepatitis B liver failure (ACHBLF) [2]. It is demonstrated that pro-inflammatory and anti-inflammatory cytokines play a crucial role in the pathogenesis of ACHBLF and constantly influence the disease progression. Level of several cytokines has been found to be elevated in patients with ACHBLF, which may be led to endotoxemia, cytokine release by necrotic liver cells, and/or reduced hepatic removal [2–6]. Consequently, elevated cytokines aggravate liver injury by mediating acute systemic inflammatory response and raise the fatality rate [6]. However, the exact mechanism of ACHBLF is not fully delineated.

Peroxisome proliferator-activated receptor gamma (PPAR-γ) is a member of nuclear receptor supergene family that functions in ligand-dependent transcription [7, 8]. The PPAR-γ is abundantly expressed in adipose tissue, colon, spleen, and macrophages and is believed to play a role in adipocyte differentiation, lipid metabolism, and glucose homeostasis by exerting effect on gene transcription [9–12]. Recently, accumulating evidence has demonstrated that PPAR-γ is involved in anti-inflammatory reactions both in vitro and in vivo. Several studies have investigated the effects of PPAR-γ activation on the inflammatory responses of monocytes and macrophages. It is shown that PPAR-γ activation can inhibit the expression of the inducible nitric oxide synthase (iNOS), gelatinase B, and scavenger receptor A genes in response to PPAR-γ ligands and can also inhibit inflammatory gene expression in part by antagonizing the activities of the transcription factors AP-1, STAT, and NF-kB in transfected cell lines [13, 14]. Additionally, it is revealed that PPAR-γ functions to dampen inflammation and injury and synthetic agonists remarkably activate PPAR-γ and amplify the effect in animal models of acute lung injury [15]. Similar results are observed in colitis [16] and acute pancreatitis [17] in vivo. All these findings suggest that PPAR-γ may serve as an anti-inflammatory mediator and play a protective role in inflammatory diseases.

In our previous study, we found PPAR-γ was significantly down-regulated due to promoter hypermethylation, and its expression level was correlated with intrahepatic fibrosis and inflammation grade in patients with chronic hepatitis B (CHB) [18]. ACHBLF is quite a different situation from CHB, which is basically a transition from the chronic phase into the fulminant phase and manifests as a process of acute decompensation of liver function. Considering the widely different mechanisms underlying the two situations, it is still of great importance to examine the role of PPAR-γ in ACHBLF. Therefore, it arouses our interest to detect the change of promoter methylation status and expression of PPAR-γ gene and its possible role in the acute event.

Therefore, the current study aimed to determine the promoter methylation patterns and expression of PPAR-γ gene and plasma level of inflammatory cytokines in patients with ACHBLF to explore the role of PPAR-γ in the progression and prognosis of ACHBLF and test its possibility to be used as a biomarker for prediction and surveillance.

## Results

### General characteristics of subjects

The selection process for subjects enrolled is shown in Additional file 1: Figure S1. Eventually, 81 ACHBLF patients, 50 CHB patients, and 30 healthy controls were enrolled in the present study. The demographic and clinical characteristics are shown in Table 1.

**Table 1:** Baseline characteristics of the subjects enrolled in the study

	ACHBLF	CHB	HC
Cases (n)	81	50	30
Age (years)	44.25 ± 13.65	41.80 ± 14.04	41.57 ± 12.09
Gender (m/f)	57/24	35/15	21/9
HBeAg (+/-)	37/44	23/27	0/30
Log_10_ [HBV DNA]	5.16 ± 1.71	5.64 ± 1.64	NA
ALT (U/L)	489.45 ± 642.68*^,^**	362.92 ± 470.21*	27.10 ± 11.55
AST (U/L)	398.61 ± 534.34*^,^**	183.40 ± 202.84*	26.77 ± 7.34
TBIL (μmol/L)	296.43 ± 158.18*^,^**	30.19 ± 38.44*	12.48 ± 5.08
Cr (μmol/L)	73.35 ± 42.66*^,^**	63.34 ± 13.64*	57.33 ± 12.79
ALB (g/L)	33.48 ± 8.73*^,^**	40.86 ± 5.67*	47.00 ± 4.53
PTA (%)	37.84 ± 9.06*^,^**	91.94 ± 17.39	93.60 ± 14.17
INR	1.87 ± 0.63*^,^**	1.02 ± 0.14	1.01 ± 0.11
Ascites (%)	76.54*^,^**	24.00*	0
Encephalopathy (%)	34.57*^,^**	0	0
Mortality (%)	54.32*^,^**	0	0

Data are shown as mean ± standard deviation

*ACHBLF* acute-on-chronic hepatitis B liver failure, *CHB* chronic hepatitis B, *HC* healthy control, *HBV* Hepatitis B virus, *ALT* alanine aminotransferase, *AST* aspartate aminotransferase, *TBIL* total bilirubin, *Cr* serum creatinine, *ALB* albumin, *PTA* prothrombin activity, *INR* international normalized ratio, *NA* not available

*Versus HC *P* < 0.05; **Versus CHB *P* < 0.05

### Methylation frequency of PPAR-γ promoter in patients with ACHBLF

**Fig. 1:**
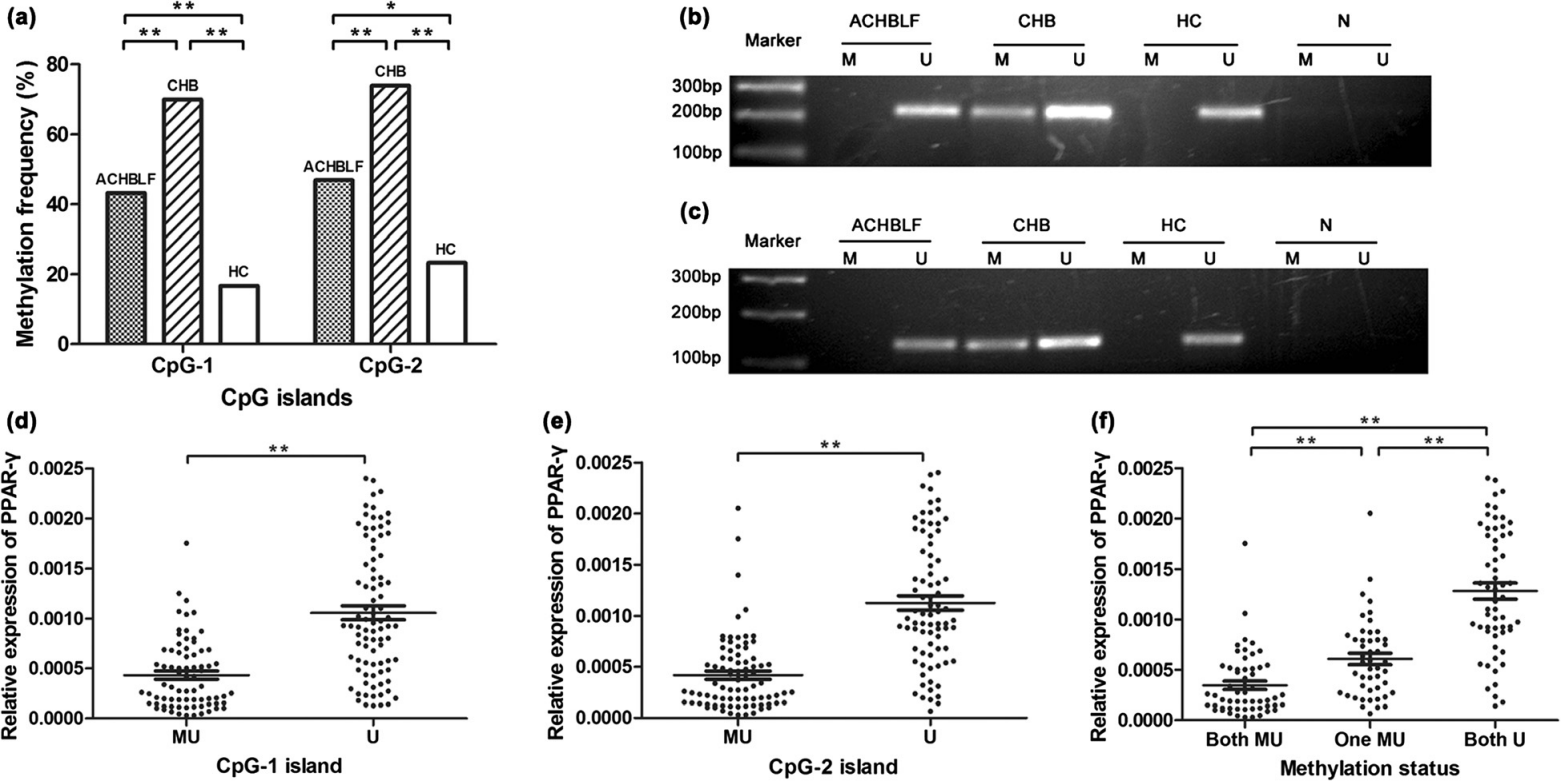
Methylation status of PPAR-γ promoter and comparison of PPAR-γ mRNA expression level. **a** The methylation frequency of PPAR-γ promoter in acute-on-chronic hepatitis B liver failure (ACHBLF) patients was significantly decreased compared with chronic hepatitis B (CHB) patients but was significantly higher than healthy controls (HC). **b** For CpG-1 island, products of methylation-specific PCR (MSP) are displayed. 218 bp M lanes and 221 bp U lanes. *M* for methylation state of CpG island on PPAR-γ promoter, *U* for unmethylation state, N for negative control. **c** For CpG-2 island, products of MSP are displayed. 138 bp M lanes and 139 U lanes. For both CpG-1 (**d**) and CpG-2 (**e**) islands, PPAR-γ mRNA expression level was significantly lower in methylated group than that in unmethylated group. **f** mRNA level was decreased stepwise as methylated CpG islands added. *P < 0.05, **P < 0.01

We first detected the methylation status of two CpG islands in PPAR-γ promoter in all enrolled subjects (Table 2). The methylation frequency of PPAR-γ promoter in ACHBLF patients was significantly decreased compared with CHB patients (CpG-1, *χ*^2^ = 8.918, *P* = 0.003; CpG-2, *χ*^2^ = 9.268, *P* = 0.002), however, was still greatly higher than healthy controls (CpG-1, *χ*^2^ = 6.691, *P* = 0.009; CpG-2, *χ*^2^ = 5.050, *P* = 0.025). Also, we found that methylation frequency of both CpG islands in CHB patients was prominently higher than healthy controls (CpG-1, *χ*^2^ = 21.333, *P* < 0.001; CpG-2, *χ*^2^ = 19.448, *P* < 0.001) (Fig. 1a), and this was identified with our former study.

**Table 2:** A cross table for methylation status of two islands in all subjects

	CpG-1 island		Total	CpG-2 island		Total
	MU	U		MU	U	
ACHBLF	35	46	81	38	43	81
CHB	35	15	50	37	13	50
HC	5	25	30	7	23	30
Total	75	86	161	82	79	161

*MU* for methylation and unmethylation states both presented, *U* for only unmethylation status presented

### Hypermethylation of CpG islands in PPAR-γ gene promoter resulted in down-regulation

Due to the fact that alteration of promoter methylation status is a usual mechanism affecting transcriptional activity, we then measured the expression level of PPAR-γ. The messenger RNA (mRNA) level of PPAR-γ in subjects with or without methylation was compared. The results showed that relative expression of PPAR-γ was significantly decreased with either CpG-1 (*Z* = −6.613, *P* < 0.001) or CpG-2 (*Z* = −7.712, *P* < 0.001) island methylation (Fig. 1c, d), and the suppression seemed to be superposable (both MU vs. one MU, *Z* = −4.085, *P <* 0.001) (Fig. 1e). The data confirmed our deduction and were basically in agreement with our previous study.

### Correlation between increased PPAR-γ mRNA level and clinical parameters in patients with ACHBLF

**Fig. 2:**
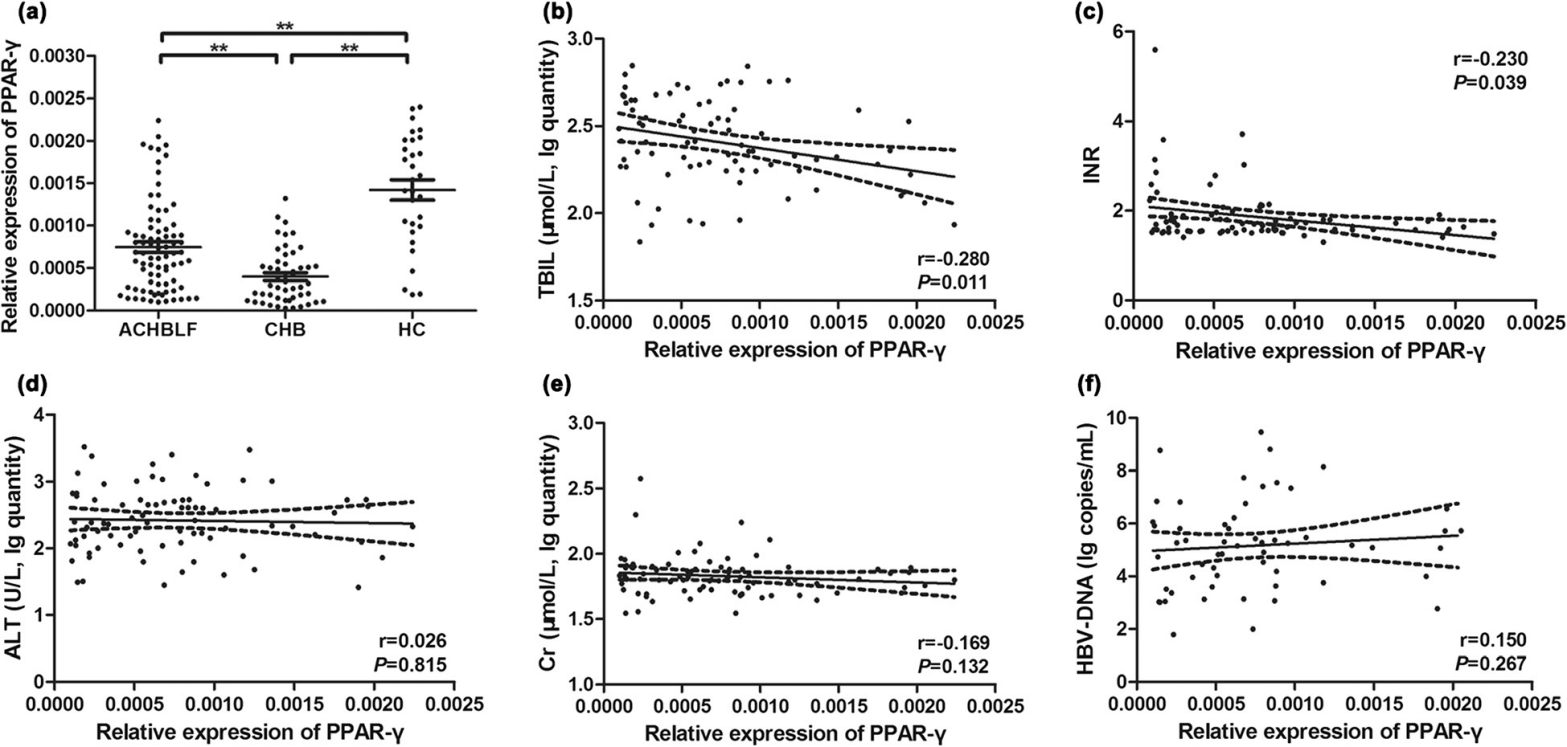
PPAR-γ mRNA level of all subjects and correlation with clinical parameters in ACHBLF patients. There was significant difference in PPAR-γ mRNA expression level between any two of the three groups (**a**). PPAR-γ mRNA expression level was negatively correlated with serum total bilirubin (TBIL) (**b**) and international normalized ratio (INR) (**c**). No significant correlations were found between PPAR-γ mRNA expression level and alanine aminotransferase (ALT) (**d**), serum creatinine (Cr) (**e**), or hepatitis B virus (HBV)-DNA load (**f**). *P* values and *r*, correlation coefficient, are shown. *P < 0.05, **P < 0.01

Moreover, we found that PPAR-γ mRNA level was significantly elevated in ACHBLF patients compared with CHB patients (*Z* = −4.003, *P* < 0.001) (Fig. 2a). However, PPAR-γ mRNA level was significantly lower in ACHBLF patients than that in healthy controls (*Z* = −4.632, *P* < 0.001). Meanwhile, expression of PPAR-γ was significantly lower in CHB patients than healthy controls (*Z* = −6.037, *P* < 0.001). These results were corresponding with the alteration of promoter methylation status we observed and showed above. To further study the role of PPAR-γ in ACHBLF, we combined with clinical data that reflected liver function of the patients. Linear correlation analysis showed that expression of PPAR-γ was negatively correlated with total bilirubin (TBIL) (*r* = −0.280, *P* = 0.011, Fig. 2c) and international normalized ratio (INR) (*r* = −0.230, *P* = 0.039, Fig. 2d). No significant correlation was found between PPAR-γ mRNA level and alanine aminotransferase (ALT), creatinine (Cr), or HBV-DNA load in patients with ACHBLF (Fig. 2b, e, f). These data suggested that PPAR-γ was closely related with the severity of ACHBLF.

### Level of cytokines and relation with PPAR-γ promoter methylation in patients with ACHBLF

**Fig. 3:**
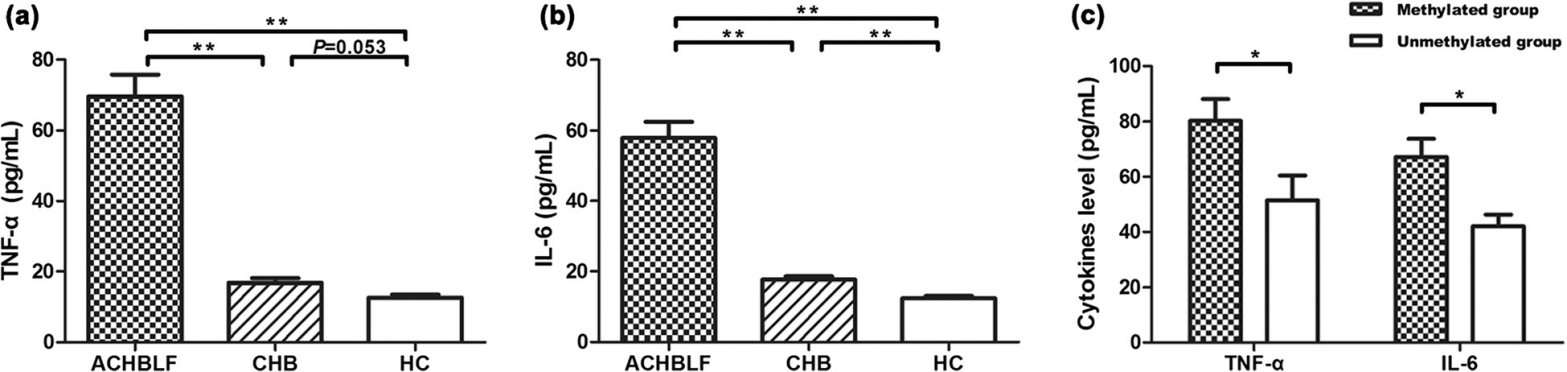
Plasma level of cytokines in all subjects and comparison between different groups in ACHBLF patients. Level of both TNF-α (**a**) and IL-6 (**b**) was significantly increased in ACHBLF patients than chronic hepatitis B (CHB) patients and healthy controls. **c** In patients with ACHBLF, level both TNF-α and IL-6 was significantly higher in methylated group (defined as either CpG island was methylated, *n =* 51) than those in unmethylated group (defined as neither CpG island was methylated, *n =* 30). *P < 0.05, **P < 0.01

Now that PPAR-γ was shown to be involved in the disease, we tried to further study the probable mechanism. The plasma level of TNF-α and IL-6 was assessed by enzyme-linked immunosorbent assay (ELISA) in the subjects. Level of both cytokines was significantly increased in ACHBLF patients compared with CHB patients (TNF-α, *t* = 6.649, *P* < 0.001; IL-6, *t* = 6.784, *P* < 0.001) and healthy controls (TNF-α, *t* = 5.582, *P* < 0.001; IL-6, *t* = 5.961, *P* < 0.001) (Fig. 3a, b). No significant difference in level of both cytokines was found between CHB patients and healthy controls. In patients with ACHBLF, level of plasma cytokines in methylated group was significantly higher than unmethylated group (TNF-α, *t* = 2.312, *P* = 0.023; IL-6, *t* = 2.639, *P* = 0.012) (Fig. 3c). These results implied that relative elevation of PPAR-γ expression as a result of promoter demethylation could effectively repress the pro-inflammatory cytokines.

### Association between PPAR-γ promoter methylation and prognosis of patients with ACHBLF

**Fig. 4:**
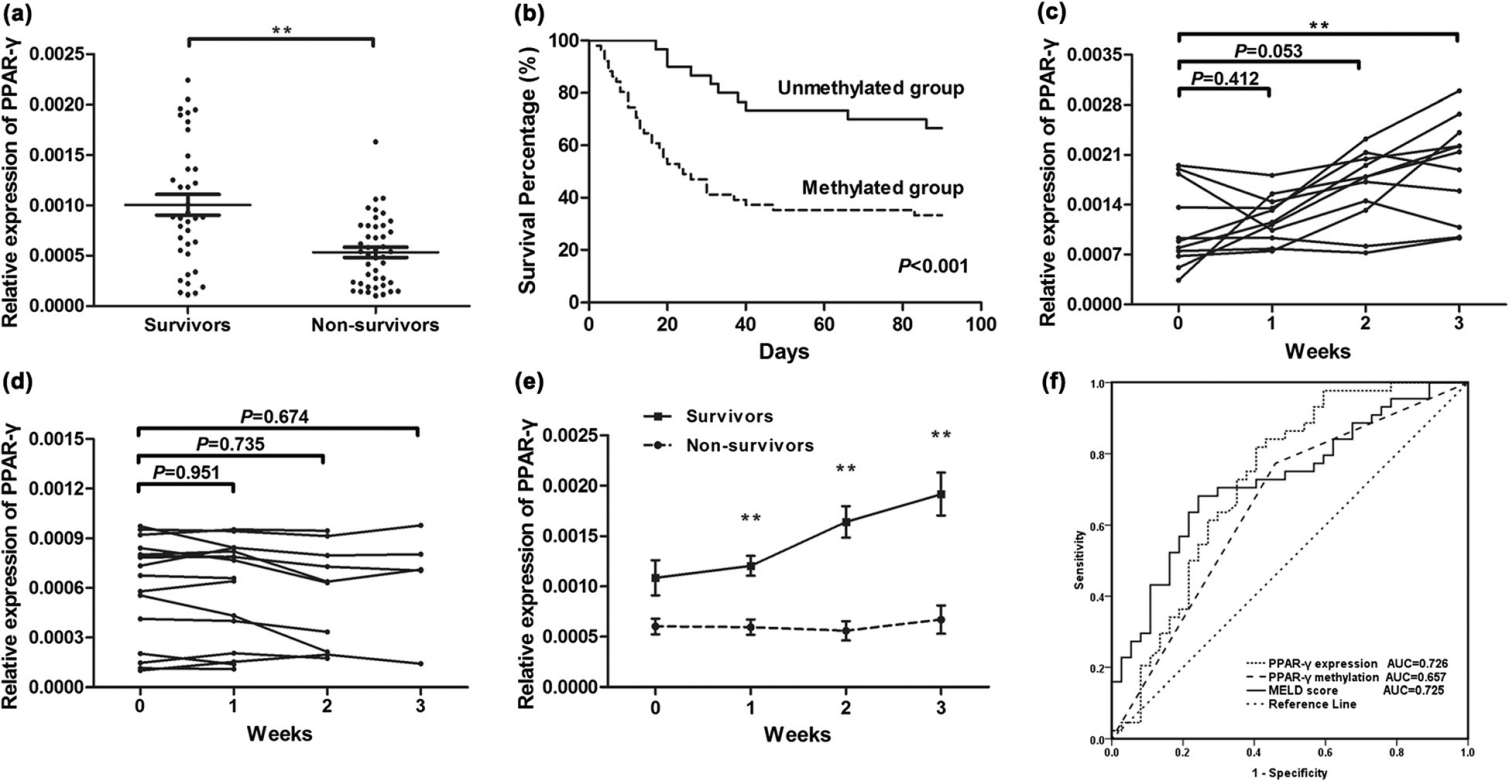
PPAR-γ mRNA level and promoter methylation predict outcomes of ACHBLF patients. **a** The mRNA level of PPAR-γ in survivors (*n =* 37) was significantly higher than that in non-survivors (*n =* 44). **b** Kaplan-Meier graph showed that survival probability of unmethylated group (*n =* 30) was higher than methylated group (*n =* 51) in patients with ACHBLF. Serial PPAR-γ mRNA expression level in part of survivors (*n =* 11) (**c**) and non-survivors (*n =* 16) (**d**). **e** An overview of dynamic change of PPAR-γ mRNA expression level in part of survivors (*n =* 11) and non-survivors (*n =* 16). **f** The receiver operating characteristic (ROC) curves of PPAR-γ expression level, PPAR-γ promoter methylation, and MELD score for predicting 3-month mortality of patients with ACHBLF are shown (*n =* 81). The areas under the ROC curve (AUC) are noted. *P < 0.05, **P < 0.01

Considering the anti-inflammatory effect of PPAR-γ, we speculated that PPAR-γ might alleviate the fulminant inflammatory injury and thus prolong the survival time of ACHBLF patients. So, a short-term follow-up was conducted. The 3-month mortality was 54.32 % (44/81), and the mean survival time is 53.47 (SE 4.057, 95 % CI 45.39–61.54). The PPAR-γ mRNA level of survivors was significantly higher than non-survivor (*Z* = −3.489, *P* < 0.001, Fig. 4a). Kaplan-Meier survival curve for ACHBLF patients with or without PPAR-γ methylation was demonstrated in Fig. 4b. Prognosis of methylated group was significantly poorer compared with unmethylated group (*χ*^2^ = 11.140, *P* < 0.001). The mean survival time for methylated group and unmethylated group was 42.24 days (SE 5.113, 95 % CI 31.97–52.50) and 72.57 days (SE 5.101, 95 % CI 62.13–83.00), respectively. A continuous surveillance of PPAR-γ mRNA expression level was carried out in twenty-seven ACHBLF patients within the first 3 weeks after admission. Data of some non-survivors were incomplete due to the intermediate death. We noted that PPAR-γ expression was significantly increased with time extending in survivors (Fig. 4c), while remained low level in non-survivors (Fig. 4d). The disparity between PPAR-γ mRNA level of the two groups was expanded week by week (Fig. 4e). Because of the significant relation between PPAR-γ and prognosis of the patients, we examined its potential use in clinical prediction. Prognostic performance was evaluated, and the receiver operating characteristic (ROC) curve for PPAR-γ methylation, PPAR-γ mRNA, and model for end-stage liver diseases (MELD) score was illustrated (Fig. 4f). The areas under the ROC curve (AUC) were 0.726 (SE 0.059, 95 % CI 0.611–0.841), 0.657 (SE 0.062, 95 % CI 0.535–0.778), and 0.725 (SE 0.056, 95 % CI 0.615–0.836), respectively.

## Discussion

In this study, we firstly reported the alteration of PPAR-γ promoter methylation status and gene expression in peripheral blood mononuclear cells (PBMCs) in patients with ACHBLF. We found that promoter methylation frequency was decreased and mRNA level of PPAR-γ was elevated in ACHBLF patients when comparing with CHB patients. Plasma level of TNF-α and IL-6 prominently rose in patients with ACHBLF and both cytokines level were lower in unmethylated group than methylated group. Moreover, promoter methylation status and mRNA level of PPAR-γ were closely associated with prognosis of patients with ACHBLF in the 3-month follow-up. The unmethylated group, which also meant the group with higher PPAR-γ expression level, showed a better outcome than the methylated group. Dynamic observation showed that PPAR-γ expression level was elevated in survivors while remained low level in non-survivors.

CpG islands refer to DNA segments which are abundant in CpG content, and CpG islands usually locate in the promoters [19, 20]. Methylation of CpG islands in gene promoter is an important regulating pattern of gene expression and usually results in gene silencing [21]. In our previous study, we had already found that hypermethylation of promoter was an important mechanism in PPAR-γ gene silencing and synchronous methylation of the two CpG islands might exert more powerful inhibition of gene transcription. The present data provided further confirmation of the conclusion. Meanwhile, the decreased methylation frequency was corresponding with the elevated PPAR-γ expression level in ACHBLF patients.

We had known that PPAR-γ expression was significantly down-regulated due to the hypermethylation occurred in promoter of CHB in our former study. Compared with CHB patients, we found that mRNA level of PPAR-γ was elevated in ACHBLF patients. However, the rebound was limited and failed to reach the normal level in comparison with health controls, which meant the inhibitory effect still existed with HBV infection. This phenomenon revealed the immune system disorder in ACHBLF and could be one of the mechanisms which contributed to the onset and exacerbation of the disease.

Although the pathogenesis of ACHBLF still remains to be elucidated, the important role played by cytokines has been long focused. It is believed that imbalanced expression of pro-inflammatory and anti-inflammatory cytokines may contribute to immunopathogenesis in ACHBLF. Among them, TNF-α and IL-6 are two key cytokines. It was reported that TNF-α production was increased in acute liver failure (ALF) [22, 23]. Zou et al. found that intrahepatic expression of TNF-α was closely correlated with the intrahepatic CD4^+^ CD8^+^ T lymphocytes and Kupffer cells (KCs) cellular infiltration in ACHBLF [5]. It has also been shown that TNF-α level in ALF correlated subsequent serious clinical complications, such as infection and encephalopathy [24–26]. Gazzard et al. reported elevated IL-6 level with multiple organ failures and increased mortality in ALF [27]. A recent study found that IL-6 may be involved in ACHBLF by upregulating Th17 response which mediates massive tissue inflammation by recruiting immune cells [28]. Moreover, it has been reported that activation of PPAR-γ could inhibit production of inflammatory cytokines by monocytes [14]. In agreement with the above studies, we found that plasma level of TNF-α and IL-6 in ACHBLF patients were dramatically higher than CHB patients and healthy controls, and level of both cytokines declined in unmethylated group compared with methylated group, which meant elevation of PPAR-γ expression level could lead to decreasing of pro-inflammatory cytokines. Taken together, we speculated that PPAR-γ gene expression elevation due to the attenuation of promoter methylation could be a self-protective measure under inflammatory stress by down-regulating level of TNF-α and IL-6 which were involved in immune-mediated liver injury in ACHBLF.

Therefore, PPAR-γ is supposed to be closely related with prognosis of patients with ACHBLF, which was confirmed by our 3-month follow-up. Patients without PPAR-γ methylation had better chances to survive. PPAR-γ mRNA level was higher and continuously elevated in survivors, while lower and sustained at low level in non-survivors with time extending. Thus, we evaluated the performance of PPAR-γ to predict prognosis of patients with ACHBLF. Although PPAR-γ methylation (AUC = 0.657) was poorer than MELD score (AUC = 0.725), PPAR-γ expression (AUC = 0.726) was comparable to MELD score and could be regarded as a valuable prognostic marker. Still, more studies focusing on the prognostic performance of PPAR-γ are needed to verify the conclusion.

According to the Asian Pacific Association for the Study of Liver (APASL) practice guideline, inhibition of the inflammatory cytokines might offer a novel approach for reducing the morbidity and mortality in patients with ACHBLF due to the fact that cytokines influence the development and course of ACHBLF [2]. Our study suggested that PPAR-γ might serve as a candidate treatment target for the possibility that activation of PPAR-γ could down-regulate pro-inflammatory cytokines and improve the prognosis of patients with ACHBLF. Chemically synthesized PPAR-γ agonists, such as thiazolidines, can selectively activate PPAR-γ to increase the peripheral sensitivity to insulin and reduce the blood glucose level and are commonly used to treat type 2 diabetes. Recently, increasing investigations have been focused on therapeutic value of PPAR-γ agonists in inflammatory diseases treatment. Suh et al. reported that PPAR-γ ligands inhibited epithelial inflammatory response in animal models of colitis, and Bordji et al. reported the anti-inflammatory properties of PPAR-γ ligands in ameliorating the cytokine-induced damage to cartilage associated with arthritis [29, 30]. Therefore, it is likely that PPAR-γ agonists are of therapeutic value to some extent in ACHBLF treatment. Despite all this, more investigations are needed to confirm the conjecture.

There are some limitations in this study. Firstly, our sample size is relatively small, which may cause a low Spearman’s rho value in the correlation analysis. However, ACHBLF is a sever condition with high mortality but low morbidity which makes it difficult to recruit a large number of patients rapidly, and the low Spearman’s rho value is attributed to not only relative number of patients but also other multiple factors including the basal liver function, heterogeneity of underlying diseases, and hepatic compensative capacity, which would greatly affect the alteration of clinical parameters. Still, more studies with a large scale in different cohorts are expected to validate our results. Secondly, as discussed above, in vivo and in vitro experiments with PPAR-γ agonists intervention are imperative to explore the therapeutic value of PPAR-γ agonists in ACHBLF.

The interpretation is very interesting when retrospecting the findings of our former study. PPAR-γ was believed to be involved in hepatic stellate cells (HSCs) activation and differentiation which was a core event in liver fibrosis and was a pivotal gene to maintain the adipogenic phenotype characteristics of HSCs and prevent the initiation of fibrosis [31]. Our former study also confirmed that silencing of PPAR-γ due to the promoter hypermethylation contributed to the onset and progression of liver fibrosis in CHB patients. Whereas, another important part of PPAR-γ was demonstrated in our present study that it was actually involved in anti-inflammation reactions by repressing level of pro-inflammatory cytokines such as TNF-α and IL-6 and was a protective factor in ACHBLF. So, the predominant biological functions of PPAR-γ in different diseases were indeed various and crucial. Furthermore, a recent study has revealed that PPAR-γ seems to be involved in the epigenetic regulation of miR-122 whose gene transcription is enhanced as a result of increased affinity of PPAR-γ and retinoid X receptor alpha (RXRα) complex to the gene promoter in hepatocellular carcinoma cells [32]. In turn, PPAR-γ could also be regulated in a microRNA-dependent way [33]. It is demonstrated that down-regulation of miR-132 can promote the formation of an epigenetic repressor complex inhibiting PPAR-γ expression and therefore promoting liver fibrosis in vivo [34]. These new findings suggest an exquisite role of PPAR-γ in epigenetic regulation in the liver diseases and provide new promising fields in which more mechanisms could be shed light on and better efforts to improve clinical practice might be made.

## Conclusions

In conclusion, we found decreased methylation frequency and elevated PPAR-γ expression level of PPAR-γ promoter in ACHBLF patients compared with CHB patients. Promoter methylation and expression level of PPAR-γ were closely associated with severity and prognosis of ACHBLF. Also, PPAR-γ may have a potential role in improving prognosis of patients with ACHBLF by down-regulating TNF-α and IL-6.

## Methods

### Patients and controls

Eighty-one patients with ACHBLF, 50 patients with CHB, and 30 healthy controls were recruited from July 2010 to January 2015 at Department of Hepatology, Qilu Hospital of Shandong University. Patients with ACHBLF were defined according to the consensus recommendation of the APASL [2]. To be specific, patients with acute hepatic insult manifesting as jaundice (serum bilirubin ≥5 mg/dl [85 μmol/L]) and coagulopathy (INR ≥1.5 or prothrombin activity <40 %), complicated within 4 weeks by ascites and/or encephalopathy based on CHB, were considered eligible. Patients with CHB were defined as presence of hepatitis B s antigen (HBsAg) in serum last for more than 6 months, and there was histological evidence of chronic hepatitis, according to the 2009 update of American Association for the Study of Liver Diseases (AASLD) practice guidelines for management of chronic hepatitis B [35]. Patients were excluded if they met any of the following criteria: (1) co-infected with HCV or HIV, (2) suffered from other liver diseases such as autoimmune hepatitis and alcoholic hepatitis, (3) receiving antioxidant agent or interferon therapy, (4) pregnant, and (5) complicated with hepatocellular carcinoma. A 3-month follow-up was conducted, and the outcomes were recorded in patients with ACHBLF. The start date was defined as the date of diagnosis of ACHBLF after hospital admission. There was no ACHBLF patient received liver transplantation in the study. Prior to sample collection, informed consent was obtained from each participant, and the study was approved by the local Ethical Committee of Qilu Hospital of Shandong University.

### Plasma collection and PBMCs isolation

Five milliliters of venous peripheral blood was collected from each subject, using EDTA as anticoagulant agent. Plasma was collected after centrifugation and stored at −80 °C for detection of cytokines. PBMCs were isolated by gradient centrifugation via Ficoll-Paque (Pharmacia Diagnostics, Uppsala, Sweden) according to the manufacturer’s protocol and were stored at −20 °C until use.

### DNA and RNA extraction from PBMCs

Genomic DNA was extracted from PBMCs using QIAamp DNA Blood Mini Kit (QIAGEN, Valencia, CA, USA) following the protocol provided by manufacturer and stored at −20 °C. Total RNA was extracted by phenol-chloroform-isopropanol method, purified, and resuspended in 20 μL of RNase-free water.

### Sodium bisulfate modification and MSP

The extracted DNA was treated with sodium bisulfite using an EZ DNA methylation Kit (Zymo Research, Orange, CA, USA) and then subjected to methylation-specific polymerase chain reaction (MSP). Four pairs of primers were used specifically to detect the PPAR-γ gene promoter methylation status. The conditions for PCR amplification were 40 cycles consisting of 30 s at 95 °C for denaturation, 40 s at 54.5 °C (CpG-1 island), or 52 °C (CpG-2 island) for annealing and 30 s at 72 °C for extension. The PCR products were separated via a 2.0 % agarose gel and visualized under UV illumination after staining with ethidium bromide. Primers were designed based on the upstream sequence of PPAR-γ gene [GenBank: NC_000003.12 (12287850-12471013)] (Additional file 2: Figure S2), which were described in our previous study [18] and listed in Additional file 3: Table S1.

### RT-PCR

Total RNA (1 μg per reaction) was converted into complementary DNA (cDNA) with PrimerScript^™^ RT Reagent Kit (Perfect Real Time; Takara, Japan) according to the manufacturer’s instruction, with condition of 70 °C for 5 min for denaturation and 42 °C for 60 min for reverse transcription. The cDNA production was subjected to real-time quantitative PCR (RT-PCR) to detect level of PPAR-γ mRNA, and β-actin was used as the endogenous control. The RT-PCR amplification mixtures (10 μL) included 0.5 mM each primer, 10 × SYBR Green (Toyobo, Osaka, Japan) and 0.5 μL cDNA. The real-time PCR was performed as follows: the initial step was 95 °C for 30 s, followed by 40 cycles of 95 °C for 5 s, 60 °C for 30 s and 72 °C for 30 s. Data analysis was performed with the LightCycler Software 4.0 (Roche Diagnostics, Germany), and results were determined using the comparative (2^−ΔCt^, ΔCt = Ct(PPAR-γ)-Ct(β-actin)) method.

### ELISA for detection of plasma TNF-α and IL-6 level

Level of plasma cytokines was measured using Human Tumor necrosis factor α ELISA Kit and Human Interleukin 6 ELISA Kit (EIAab, Wuhan, China), according to the standard protocols of the manufacturer. The minimum detectable dose was typically 3.9 pg/mL for TNF-α and 7.80 pg/mL for IL-6, respectively.

### Clinical parameters

Fasting venous blood was collected from each subject. Hepatitis B s antigen (HBsAg) and hepatitis B e antigen (HBeAg) were detected by an electrochemiluminescence assay (Roche Diagnostics Ltd, Mannheim, Germany). The serum level of HBV DNA was assayed by a fluorescent quantitative detection kit (Da An Gene, Guangzhou, China). ALT, aspartate aminotransferase (AST), TBIL, serum Cr, albumin (ALB), prothrombin activity (PTA), and INR were measured by routine laboratory methods. MELD scores were calculated according to the Malinchoc formula: *R* = 9.57 × ln[creatinine(mg/dL)] + 3.78 × ln[bilirubin(mg/dL)] + 11.2 × ln(INR) + 6.43 × (etiology: 0 if cholestatic or alcoholic, 1 otherwise) [36].

### Statistical analysis

All data were analyzed using SPSS 17.0 software (SPSS Inc., Chicago, IL, USA). Comparison of methylation status among different groups was analyzed by chi-square test or Fisher’s exact test. Changes in mRNA concentration were measured by Student *t* test or Mann-Whitney *U* test. Spearman test was applied for correlation analysis. Survival curves for methylated group and unmethylated group of ACHBLF patients were compared by Log-rank test. All statistical analyses were two-sided and *P* value <0.05 was considered statistically significant.

## Additional files

Additional file 1: Figure S1. Flowchart of subjects selection. The process of inclusion and exclusion of subjects is shown. (DOC 30 kb) Additional file 2: Figure S2. Schematic figure of peroxisome proliferator-activated receptor gamma (PPAR-γ) gene promoter. CpG sites are shown as short vertical lines across the horizontal line. Positions of primers for methylation-specific PCR (MSP) were noted. (TIFF 839 kb) Additional file 3: Table S1. Primers for methylation-specific PCR (MSP) and quantitative real-time PCR (RT-PCR). The sequence, production size and annealing temperature are shown. (DOC 31 kb)

## Abbreviations

ACHBLF: acute-on-chronic hepatitis B liver failure
ALB: albumin
ALT: alanine aminotransferase
AST: aspartate aminotransferase
CHB: chronic hepatitis B
Cr: creatinine
HBeAg: hepatitis B e antigen
HBsAg: hepatitis B s antigen
HBV: hepatitis B virus
INR: international normalized ratio
MELD: model for end-stage liver diseases
PBMC: peripheral blood mononuclear cell
PPAR-γ: peroxisome proliferator-activated receptor gamma
PTA: prothrombin activity
TBIL: total bilirubin
