# Supplementary material for: Promoter methylation status and expression of PPAR-γ gene are associated with prognosis of acute-on-chronic hepatitis B liver failure
Source: Clin Epigenetics. 2015 Oct 28;7:115. doi: 10.1186/s13148-015-0149-2 (PMC4625884; doi:10.1186/s13148-015-0149-2)
Supplement: Additional file 1: Figure S1. — Flowchart of subjects selection. The process of inclusion and exclusion of subjects is shown. (DOC 30 kb) [file 13148_2015_149_MOESM1_ESM.doc]

**Figure S1** Flowchart of subjects selection

185 subjects assessed

196 subjects screened

161 subjects enrolled

ACHBLF group

n=81

CHB group

n=50

HC group

n=30

13 ACHBLF patients excluded:

Co-infected with HCV or HIV (n=5)

Pregnant (n=1)

Hepatocellular carcinoma (n=7)

11 CHB patients excluded:

Co-infected with HCV or HIV (n=2)

Suffered from other liver diseases (n=9)

3 ACHBLF patients and 8 CHB patients with no consent excluded
