# Supplementary figures and images for: Promoter methylation status and expression of PPAR-γ gene are associated with prognosis of acute-on-chronic hepatitis B liver failure
Source: Clin Epigenetics. 2015 Oct 28;7:115. doi: 10.1186/s13148-015-0149-2 (PMC4625884; doi:10.1186/s13148-015-0149-2)

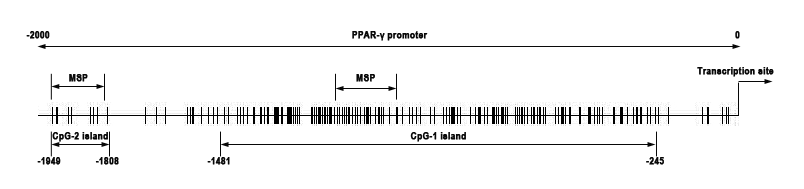

Supplement: Additional file 2: Figure S2. — Schematic figure of peroxisome proliferator-activated receptor gamma (PPAR-γ) gene promoter. CpG sites are shown as short vertical lines across the horizontal line. Positions of primers for methylation-specific PCR (MSP) were noted. (TIFF 839 kb) [file 13148_2015_149_MOESM2_ESM.tif]
