# Supplementary material for: Promoter methylation status and expression of PPAR-γ gene are associated with prognosis of acute-on-chronic hepatitis B liver failure
Source: Clin Epigenetics. 2015 Oct 28;7:115. doi: 10.1186/s13148-015-0149-2 (PMC4625884; doi:10.1186/s13148-015-0149-2)
Supplement: Additional file 3: Table S1. — Primers for methylation-specific PCR (MSP) and quantitative real-time PCR (RT-PCR). The sequence, production size and annealing temperature are shown. (DOC 31 kb) [file 13148_2015_149_MOESM3_ESM.doc]

**Table S1** Primers for methylation-specific PCR (MSP) and quantitative real-time PCR (RT-PCR)

| Primer | Primer sequence | | Production size (bp) | Annealing temperature (℃) |
| --- | --- | --- | --- | --- |
|  | Forward | Reverse |  |  |
| CpG-1 M | TATTTTTGTTGAGGAGGAGGTTTC | GACTAAAAATCCTAACTACGCGCT | 218 | 54.5 |
| CpG-1 U | TATTTTTGTTGAGGAGGAGGTTTT | TACAACTAAAAATCCTAACTACACACT | 221 | 54.5 |
| CpG-2 M | GAGTTTTATATTTCGGTTTTTTTAGATC | AACTACCTAATATCGTTTACTCCTCG | 138 | 52.0 |
| CpG-2 U | GGAGTTTTATATTTTGGTTTTTTTAGATT | AACTACCTAATATCATTTACTCCTCACC | 139 | 52.0 |
| RT-PCR | TTGTTCCAGGGAAATTCACTGC | CGCCGTAAATTATTTCTAAACC | 171 | 60.0 |
